# Modeling Morquio A Syndrome: An Anthropometric Study of Body Characteristics and Stature

**DOI:** 10.3390/diagnostics10020116

**Published:** 2020-02-20

**Authors:** Agnieszka Różdżyńska-Świątkowska, Krzysztof Szklanny, Jolanta Marucha, Anna Tylki-Szymańska

**Affiliations:** 1 Anthropology Laboratory, Children’s Memorial Health Institute, 04-730 Warsaw, Poland; 2 Multimedia Department, Polish-Japanese Academy of Information Technology, 02-008 Warsaw, Poland; kszklanny@pjwstk.edu.pl; 3 Department of Paediatrics, Nutrition and Metabolic Diseases, Children’s Memorial Health Institute, 04-730 Warsaw, Poland; jolamarucha@onet.eu (J.M.); a.tylki@ipczd.pl (A.T.-S.)

**Keywords:** Morquio A syndrome, mucopolysaccharidosis IVA, anthropometric measurements, model of Morquio A

## Abstract

Background: Morquio A syndrome or mucopolysaccharidosis (MPS) IVA is an autosomal recessive, life-limiting lysosomal storage disease caused by deficient activity of the enzyme galactosamine-6-sulfatase. Common early symptoms such as abnormalities of body stature can facilitate timely diagnosis. This study aimed to create a pattern of face and body stature based on anthropometric measurements taken from a cohort of Polish patients with MPS IVA. Methods: Analysis of 11 somatometric and 14 craniofacial features was performed on 20 patients with MPS IVA, aged from 3 months to 26 years. The diagnosis of MPS IVA was confirmed by enzymatic and molecular analysis. Two-tailed t-tests were used to compare mean values for body length and weight at birth between the MPS IVA patients and the general population. To show the degree and direction of deviation z-scores were calculated and then used to construct a model of an average MPS IVA patient. Results: Mean values for body height and weight at birth were greater for boys than for the general population. The observed pattern of head and body shape indicated that dwarfism occurred with age as a result of the relatively short trunk and lower limbs. Skeletal abnormalities included a bell-shaped chest with the ratio of chest depth to chest width being significantly above the norm. The head and neck were relatively elongated, in comparison to body height, and tucked between narrow shoulders. The head had dolichocephalic shape, while the nose was short with wide nostrils. Conclusions: Multiple anthropometric measurements, including age ranges, allowed for the creation of a model that showed the most characteristic features of the MPS IVA phenotype.

## 1. Introduction

Mucopolysaccharidosis type IVA (Morquio syndrome A, OMIM MPS IVA 253000) is an autosomal recessive disorder caused by the deficiency of the lysosomal enzyme, N-acetylgalactosamine-6-sulfatase (GALNS; 612222). In the absence of enzyme activity, stepwise degradation of keratan sulfate and chondroitin-6-sulfate is prevented, resulting in intracellular accumulation of these glycosaminoglycans (GAGs) into the lysosomes, leading to a progressive disorder with multiple tissue and organ involvement [1,2]. The incidence of MPS IVA is approximately 0.22 per 100,000 births (range 0.07 to 1.32) [3,4,5]. Over 70% of patients affected with MPS IVA show initial clinical manifestations within the first 2–3 years of life, although formal diagnosis is usually made approximately two years later [6]. Patients with MPS IVA are severely affected in growth; their final adult height can be used as an indicator of disease severity. The phenotypic spectrum for MPS IVA ranges from a rapidly progressive, early-onset classical form, to a slow-progressive late-onset non-classical form [7].

MPS IVA is characterized by unique skeletal manifestations such as disproportional dwarfism, cervical instability, thoracic deformity, genu valgum, and laxity of joints. In addition, because of systemic accumulation of GAG, patients progressively develop non-skeletal manifestations including respiratory disease, spinal cord compression, cardiac disease, impaired vision (corneal clouding), and hearing loss. Although intelligence remains normal and there is no direct central nervous system (CNS) involvement, the skeletal changes may result in the secondary peripheral nervous system (PNS) complication [1,2]. The irregularities observed in the studied patients were caused by disrupted development of bone and cartilage, most probably beginning in the fetal phase [8].

Because of the rarity of the disease and the wide spectrum of disease severity, early diagnosis is difficult. Constructing a model of face shape and body stature of an MPS IVA patient can assist a more efficient diagnosis. This study aimed to construct a drawing, obtained by using the patient’s photos, and the pattern profile obtained by z-scores.

## 2. Material and Methods

Between 1988–2018, a mix-longitudinal retrospective growth study was performed at at the Children’s Memorial Health Institute (CMHI). The study was conducted at CMHI on a group of 20 patients with the classical form of MPS IVA (7 girls and 13 boys) aged from 3 months to 26 years (mean age: 9 years; SD 10.7). All the patients had MPS IVA diagnosis confirmed by enzymatic and molecular analysis. Table S1 in the Supplementary Data presents clinical characteristics and responsible mutations of each patient. The criteria for classical form were a major reduction of body height (z-scores lower than 3) highly compromised walking ability, and the use of a wheelchair. No patients had received enzyme replacement therapy. Ethics approval and consent to participate: the protocol was approved by the human-subject institutional review board at the Children’s Memorial Health Institute (CMHI), Warsaw, Poland. Consent for publication: written informed consent was provided for all subjects, either personally or, if under the age of 18, by their parents or guardians.

Birth body length and weight values were taken from the children’s personal health records. The mean birth body height and weight were calculated. Each patient was measured a few times (ranging from 1 to 13 times) while monitoring was performed. The number of measurements for each parameter ranged from 13 to 15, and the time between measurements ranged from 6 months to 9 years. A Wolański liberometer (a type of infantometer accurate to 1 mm) was used to measure the supine length of children under 3 years. A stadiometer (accurate to 1 mm) was used to measure the standing height of the older children. In the case of serious genu valgum, segmental height in a supine position was measured. Weight was measured using an electronic scale accurate to 0.05 kg. A non-stretchable tape was used to measure head and chest circumference (accurate to 5 mm). Anthropometric measurements were obtained from each subject (Table 1) following standard anthropometric techniques [9,10]. Measurements were recorded to the nearest millimeter using standard and calibrated equipment: GPM (Gneupel Präzisions-Mechanik) sliding and spreading, blunt-ended calipers, and an anthropometer. The following parameters were obtained: somatometric and craniofacial widths, lengths, depths, heights, as well as the details of eye, nose, and mouth structure. Each parameter was performed two times and if a difference between measurements exceeded 0.3 cm, a third measurement was performed.

Statistical analysis was performed using Statistica, v.8 (StatSoft, Krakow, Poland). Shapiro–Wilk and Kołmogorov–Smirnov tests were used to assess sample normality for each observed craniofacial and somatometric parameter. The significance level was assumed at 0.05. A two-tailed t-test was used to compare the mean values of body length and weight at birth between boys with MPS IVA with Polish reference charts [11].

One of the problems with analyzing the natural history of rare diseases is the small populations within individual age groups. Therefore, age and sex data were standardized based on the mean and standard deviation of the examined feature in a given age group of healthy children using Polish reference charts [11,12,13]. This yielded z-scores showing the magnitude and direction (i.e., sign) of deviation of the evaluated data from the population mean, which is calculated according to this formula:

*z-score* = (*X* − *X_m_*)/*sd*

where: *X_m_* is the mean value of an observed parameter in the population, *sd* is the standard deviation of an observed parameter in the population.

Mean z-scores were calculated for all patients, to model an average MPS IVA patient.

Because of a different range of growth in the next stage of development in the healthy population [11,14], mean z-scores were calculated for gender groups in three age classes: before a growth spurt, below 9 years; during a growth spurt, from 10–15 years, and after a growth spurt, at 16 years and over.

A 3D model of an MPS IVA patient was constructed based on 20 photos of all MPS IVA patients (aged from 3 to 32 years). The model was created with ZBrush, currently the most popular 3D character modeling tool [15,16]. The model was evaluated by two expert computer technicians, and appropriate corrections were made. Skin texture was applied, two-point lighting was set, and the final model was rendered. Colour correction was prepared in Adobe Photoshop CC 2015 [17].

## 3. Results

### 3.1. Anthropometric Measurements at Birth

Mean values for birth length and weight are presented in Table 2. Only data from 14 patients (10 boys and 4 girls) were available due to paucity of the children’s health records. For boys with MPS IVA, mean values for birth length and weight were calculated. Birth length in boys was significantly greater than in the general Polish population [11]. Differences in mean values for birth weight were not significant (Table 2).

### 3.2. Anthropometric Assessment

**Figure 1:**
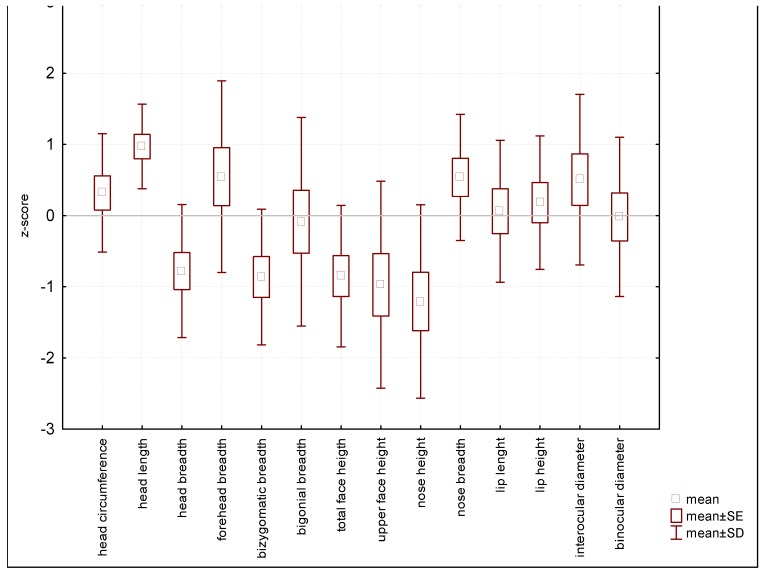
Mean values of somatometric features.

The boxplot method was used to show data. The mean height of patients with MPS IVA was shorter than in their healthy peers (−6.53 z-score), reflecting short trunk (−4.01 z-score) and very short lower limbs (−5.29 z-score). The length of the head and neck was below the 3^rd^ percentile, although it was less reduced (−2.49 z-score) in comparison to the body height mean z-score (−6.53). Skeletal abnormalities included narrow shoulders (−3.95 z-score), and narrow and convex chest (chest width was −2.54 z-score while chest sagittal diameter was 1.59 z-score) (Figure 1). Narrow shoulders are a result of abnormally shaped acromions of the scapulas and this is a characteristic feature for all mucopolysaccharidoses. The calculated mean z-score for the ratio of chest depth to chest width was 3.61.

**Figure 2:**
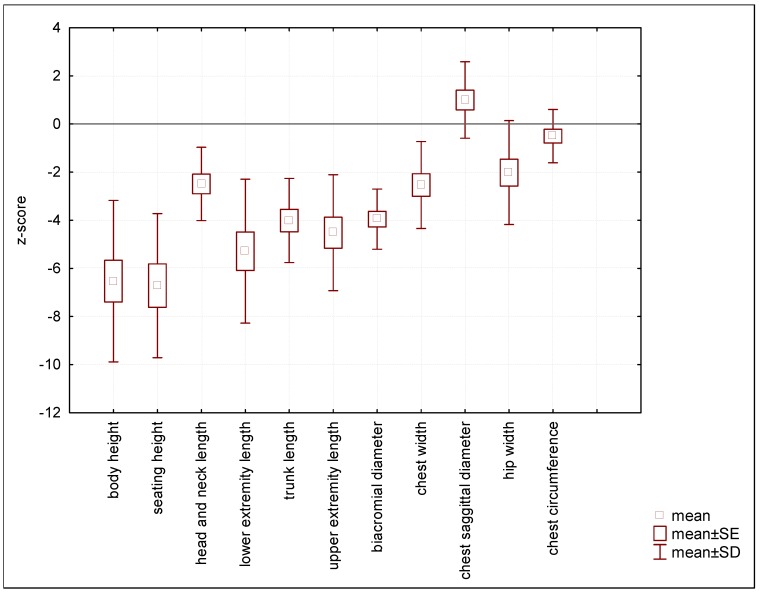
Values of craniofacial features.

Craniometric analysis showed that head circumference did not differ from healthy peers (0.32 z-score), and was appropriate for calendar age. However, in comparison to body height, head circumference was above the 97th percentile (2.57 z-scores). The head has a tendency to be slightly longer (0.97 z-score) and narrower (−0.78 z-score). Measurements for total face height were a little bit shorter (−0.86 z-score) than in the general population as well as upper-face height (−0.97 z-scores). Also, the nose was short (−1.21 z-score), with a relatively wide nostril (0.59 z-score) (Figure 2.).

**Figure 3:**
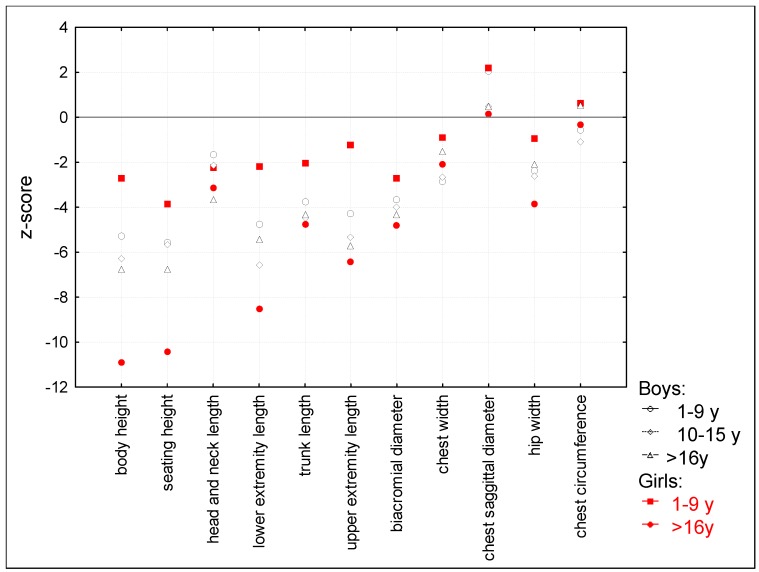
Somatometric pattern profile in three age groups.

Analysis of craniofacial and somatometric patterns was performed for each gender in three age classes. Reduced body height was observed in all age groups from −2.72 z-scores in young girls to −10.88 z-scores in older girls, and the deviation from norms increased with age (Figure 3).

**Figure 4:**
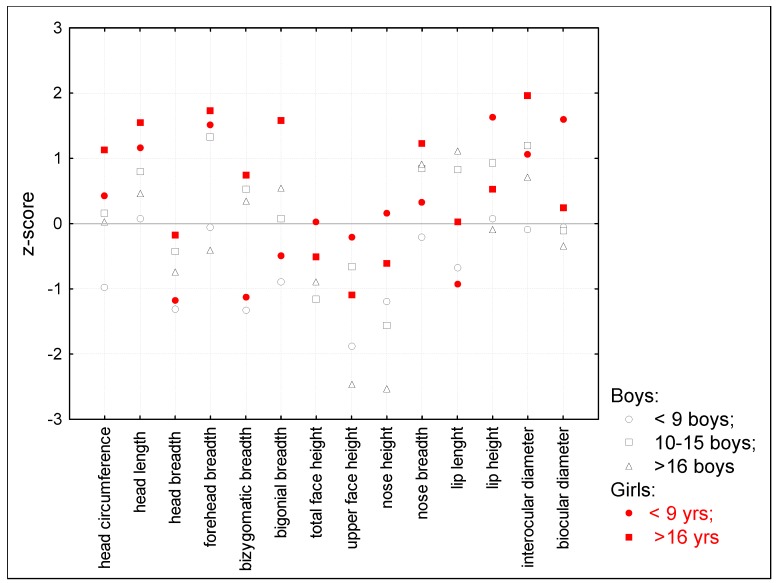
Craniofacial pattern profile in three age groups.

Also for other measurements such as sitting height, head and neck length, trunk length, and upper extremities length the deviations increased with age. In all groups, head and neck length were relatively elongated in comparison to body height. Short trunks, narrow shoulders, and convex chests were characteristic for the study group patients. In the craniofacial pattern profiles, the mean z-score range between groups was wide, but there were the same tendencies for all age groups such as a narrow but elongated head, reduced total face height, and a short nose with wide nostrils (Figure 4).

**Figure 5:**
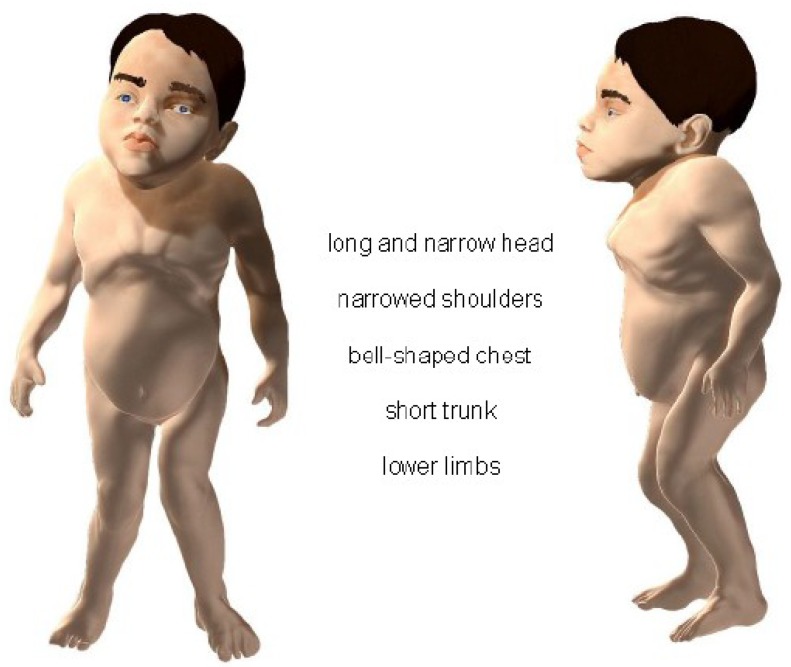
Computer profile.

Tables S2 and S3 present the values of mean z-scores for observed features. A 3D model of an MPS IVA patient is presented in Figure 5.

## 4. Discussion

Creating a face shape and body stature model can be a tool for early diagnosis of rare disorders such as Morquio A and other mucopolysaccharidoses. The model in this study was constructed according to measurements and z-score estimates of the highest possible precision, thereby allowing the accurate rendering of both the face and body. Mathematical modeling in science is primarily employed to simplify certain complex processes. Modeling allows the scale of the phenomenon to be changed, thus enhancing understanding. In the case of biology, modeling performs a particular role, enabling the separation of complicated relationships and processes. A good model can replace a series of individual measurements performed on a small number of parameters describing individual development. Creating a universal model of the body stature of MPS IVA is a challenging task because of the wide spectrum of disease severity. There is a scarcity of literature reporting detailed anthropometric data for children with MPS diseases [6,7]. Constructing such a model requires measurement tools of maximum precision and monitoring of development parameters, specifically age ranges. This mixed-longitudinal study provided the possibility to investigate the physical development of patients with MPS IVA. The results showed that anthropometric features of MPS IVA patients differed from the healthy population. Mean body length for boys in the studied MPS IVA group was statistically significantly greater than the healthy population at the time of birth. This trend was corroborated in previous publications; it is also characteristic in other mucopolysaccharidoses including MPS I, II, and VI [18,19,20]. The pathomechanism of this phenomenon is still unclear. Following birth, children with MPS IVA grow slowly and reach their final height at approximately eight years. This corresponds to a −8 z-score for height in healthy peers [6]. In our study, patients stop growing on average at approximately 7.6 years. The mean z-score for body height was −6.53, but for groups, there was a difference between the oldest groups for boys and girls, with a mean z-score for boys of −6.76, and −10.9 for girls. The reason for this difference was unknown, we could not rule out the influence of unknown external factors. Doherty describes a case of a patient who reached a maximum adult height of 86.4 cm; this corresponds to a –13 z-score compared to nationally published reference charts, and his phenotype was more severe than an average MPS IVA patient [21].

The study showed that differences in body proportion between healthy and affected children increased with age. Analysis of anthropometric measurements among MPS IVA patients allowed for the distinction of features that deviated from the normal population. The thoracic spine from T1 to T12, as well as the lumbar region of the spine from L1 to L5, stop growing early, causing MPS IVA patients to have a short trunk [7]. The most common deformity of the lower extremities in MPS IVA patients presents the knee and ankle valgus. The articular cartilage is erosive and irregular and is degenerated quickly, leading to the build-up of early arthrosis, especially in the lower extremities. In this study, a significant reduction of body height was observed in all patients because of a short trunk and short lower extremities [22]. The shoulders were narrow and the chest presented pectus carinatum deformities. This is a marked deformity of the anterior chest wall caused by rib overgrowth, compared to other parts of the body, which restricts the lungs [7]. Because of degenerative changes in body stature, MPS IVA may be misdiagnosed as spondyloepiphyseal dysplasia or other musculoskeletal disorders [23], however, a typical characteristic for MPS IVA is the presence of hypermobile joints secondary to ligamentus laxity [24]. Individuals with MPS IVA are described as having large heads, in actual fact, the head is large in comparison to body height but normal for the patient’s age, because the frontal bone, parietal bone, occipital bone, and mandible bone grow for a longer time in comparison with the body. This causes a prominent forehead and elongated head [21]. In our study, head and neck lengths were relatively long in comparison to body height. This is due to MPS IVA patients having shorter necks, as the cervical spine in the C1–C7 region stops growing earlier than the head [7]. Notwithstanding this, the head has a tendency for vertical growth, leading to a dolichocephalic facial shape [25]. Overall, the imbalance of growth is the most characteristic feature in MPS IVA [26].

Enzyme replacement therapy (ERT) and hematopoietic stem cell transplantation are clinically available for patients with MPS IVA. Due to the progressive nature of Morquio A syndrome, early diagnosis is critical to ensure rapid treatment and optimal patient outcomes [27]. The method used in this study could be used to construct models for other rare diseases with dysmorphic features, especially other MPS.

The study could have been improved. Morquio A disease is a rare disorder and we only had a limited data sample. For rare diseases, it is difficult to collect enough data to make separate analyses for sex and age groups. A larger number of patients in the study group would be required over the next 20 years to be sufficient. Also for analysis of natural history and body proportion we need patients who have not received ERT, which will be difficult due to the availability of treatment. Therefore, some alternatives must be found. In our study to construct a universal model of body proportions for patients with MPS IVA, all measurements were standardized to age and gender. This procedure transforms two different variables (with incomparable measures) into one comparable statistical measure. In the first analysis, we pool all data together to show a universal model of body stature and face shape for individuals with MPS IVA. This has disadvantages as the image of the studied group may be imprecise, because of changes in body proportions increasing with age. Therefore, the second analysis for age groups and gender was performed. Our study has a mixed-longitudinal character [28,29]. hence, this method can be used when there is an insufficient number of subjects [6,28]. A great number of patients had problems with deformities of their lower extremities which, despite applied procedures, might have resulted in measurement errors. Nonetheless, although the modeling of the studied group may have been imperfect, the final model still demonstrated the main characteristics of MPS IVA.

## 5. Conclusions

Multiple measurements, including age ranges, allowed the creation of a model that showed the most characteristic features of the MPS IVA phenotype:

1. The results of anthropometric measurements of all the MPS IVA patients showed similar characteristics. 

2. This allowed the generation of a face shape and body stature model. 

3. This type of model, which includes body proportions and facial features of MPS IVA patients based on accurate anthropometric measurements, can assist early diagnosis.

## Figures and Tables

**Table 1:** Anthropometric measurements employed in this study.

**Craniofacial (cm)**		
Head Circumference	OFC (occipital frontal circumference)	Maximum circumference in Frankfurt plane at the level of the glabella and opistocranion
**Craniofacial (mm)**		
Head Length	g-op	Glabella to opistocranion
Head Breadth	eu-eu	Eurion to eurion
Forehead Breadth	ft-ft	Frontotemporale to frontotemporale
Bizygomatic Breadth	zy-zy	Zygion to zygion
Bigonial Breadth	go-go	Gonion to gonion
Total Face Height	n-gn	Nasion to gnathion
Upper Face Height	n-sto	Nasion to stomion
Nose Height	n-sn	Nasion to subnasale
Nose Breadth	al-al	Alare to alare
Lip Lenght	ch-ch	Chelion to chelion
Lip Height	ls-li	Labiale superius to labiale inferius
Interocular Diameter	en-en	Endocanthion to endocanthion
Biocular Diameter	ex-ex	Exocantion to exocantion
**Somatometric (cm)**		
Body Height		Basis to vertex
Seating Height		Vertex to tuberale
Head and Neck Length (v-sst)	v-sst	Vertex to suprasternale
Lower Extremity Length (B-sy)	B-sy	Basis to symphysion
Trunk Length (sst-sy)	sst-sy	Suprasternale to syphysion
Upper Extremity Length (a-daIII)	a-daIII	Acromion-dactylion III
Biacromial Diameter (a-a)	a-a	Acromion-acromion
Chest Width (thl-thl)	thl-thl	Thoracolaterale-thoracolaterale
Chest Saggittal Diameter (xi-ths)	xi-ths	Xiphoidale-thoracospinale
Hip Width (ic-ic)	ic-ic	Iliocristale to iliocristale
Chest Circumference		Circumference in the horizontal plane at the level of xiphoidale

**Table 2:** Comparison of birth length and weight at birth among boys with MPS IVA and healthy controls.

		Bodt Weight (g)	Body Length (cm)	*p* Value for Weight *	*p* Value for Length *
Study group	Boys (*n* = 10)	3719.3 ± 461	57.3 ± 3.34	0.35	0.003
Healthy population	Boys	3500 ± 600	52.2 ± 2.8		

* t-test; Bold font indicates statistically significant values.

## Supplementary Materials

The following are available online at https://www.mdpi.com/2075-4418/10/2/116/s1, Table S1: Clinical characteristics of 20 patients with Morquio IVA patients (sibilings are marked in colours), Table S2: Mean z-score±sd values for anthropometric measurements in patients with MPS IVA, Table S3. Mean z-scores±sd values for observed groups of patients with MPS IVA.

## Abbreviations

MPS	Mucopolysaccharidosis
GALNS	Galactosamine (N-acetyl)-6-Sulfatase
GAGs	Glikosaminoglycans
CNS	Central Nervous System
PNS	Peripheral Nervous System
OFC	Head Circumference
HS	Heparan Sulfate
